# Vascular endothelial growth factor expression and T-regulatory cells in premenopausal breast cancer

**DOI:** 10.3892/ol.2013.1142

**Published:** 2013-01-18

**Authors:** FRANCESCO RECCHIA, GIAMPIERO CANDELORO, STEFANO NECOZIONE, GIOVAMBATTISTA DESIDERI, ALISIA CESTA, LAURA RECCHIA, SILVIO REA

**Affiliations:** 1 Department of Oncology, Civilian Hospital, Avezzano;; 2 Carlo Ferri Foundation, Monterotondo, Rome;; 3 Department of Internal Medicine and Public Health, University of L’Aquila, L’Aquila;; 4 Department of Health Sciences, University of Molise, Campobasso;; 5 Department of Experimental Medicine, University of L’Aquila, L’Aquila, Italy

**Keywords:** vascular endothelial growth factor, T-regulatory cells, premenopausal breast cancer, chemotherapy

## Abstract

Estradiol (E2) plays a key role in human reproduction through the induction of vascular endothelial growth factor (VEGF) and T-regulatory cells (T-Regs), which are also important in breast cancer (BC) growth. The primary endpoint of the present study was the investigation of whether E2 suppression, chemotherapy and radiation therapy decreased the levels of VEGF and T-Regs of premenopausal patients with high-risk early BC. The secondary endpoints were toxicity, progression-free survival (PFS) and overall survival (OS). Between April 2003 and July 2008, 100 premenopausal women with early, high-risk BC were entered into the study. The characteristics of the patients were as follows: median age, 43 years (range, 26–45); median number of positive axillary nodes, 3.3; median Ki-67, 33%. Plasma E2, VEGF and T-Reg were measured at baseline and every year. Treatment comprised luteneizing hormone-releasing hormone (LH-RH) analogue, tailored chemotherapy, radiation therapy and hormonal therapy in oestrogen receptor-positive (ER^+^) tumours. At 4 years, a statistically significant decrease in E2, VEGF and T-Reg levels was observed; the PFS and OS rates were 94 and 98%, respectively. Hot flushes and G1 osteopenia occurred following LH-RH analogue administration, while no unexpected toxicity was observed following chemotherapy. E2 deprivation with an LH-RH analogue, tailored chemotherapy, radiation therapy and hormonal therapy in ER^+^ tumours decreased plasma VEGF levels and T-Regs numbers in premenopausal high-risk ER^+^ and ER- BC patients. In addition, a favorable impact on PFS and OS was observed.

## Introduction

Estradiol (E2) plays a fundamental physiological role in all phases of human reproduction; it is also a key hormone in breast carcinogenesis (1). E2 acts through two basic pathways involving vascular endothelial growth factor (VEGF) and T-regulatory cells (T-Regs).

In adult females, E2 modulates VEGF expression in uterine and breast cells through transcriptional activation of the oestrogen receptor (ER) (2). In the late proliferative phase of each menstrual cycle, the level of VEGF mRNA increases 3.6-fold to allow embryo implantation and basal membrane erosion if fertilisation takes place; repeated abortions occur if E2 is low (3). E2 plays essential roles in regulating blastocyst implantation, trophoblast invasiveness and remodelling of uterine arteries (4).

T-Regs are also fundamentally necessary for mammalian reproduction. During early pregnancy, the maternal immune system has to both tolerate paternal alloantigens and maintain defences against pathogens. T-Regs have the physiological function of modulating immune response-suppressing T-cell proliferation and cytokine production (5), thus preventing excessive immune reactions. In the fertile female, E2 promotes tolerance of the heterologous fertilised embryo by expanding the T-Reg compartment and inducing the transformation of peripheral CD4^+^ T cells toward a FoxP3+ T-Reg phenotype (6).

VEGF and T-Regs are also fundamental factors in breast cancer (BC) progression, and E2 is the principal inducer of both. VEGF and T-Regs may act as tumour promoters in the presence of initiated cell clones (7), and VEGF and T-Regs are reportedly correlated with response to treatment (8–10). Since E2 is the principal inducer of both VEGF and T-Regs, it is conceivable that by decreasing E2 with a luteneizing hormone-releasing hormone (LH-RH) analogue as part of the adjuvant therapy, we may decrease the levels of VEGF and T-Regs and thus improve the clinical outcome of premenopausal patients with BC.

The primary endpoint of the present study was to investigate whether administration of an LH-RH analogue, followed by chemotherapy, radiation therapy and hormonal therapy (in ER^+^ patients) would reduce VEGF and T-Reg levels in premenopausal patients with high-risk early BC. The secondary endpoints were toxicity, progression-free survival (PFS) and overall survival (OS).

## Patients and methods

### Eligibility

This multicentre, phase II, single-arm study included premenopausal BC patients of 18–45 years of age, who were treated with modified radical mastectomy or quadrantectomy, plus axillary dissection. All subjects had high-risk invasive BC of a pathological tumour stage (pT2–pT3a) with ≥1 positive axillary lymph node and no distant metastasis. Continuing ovarian function was biochemically confirmed by the following laboratory data: follicle-stimulating hormone (FSH), <10 mU/ml; LH, <0.8 mU/ml; 17β-E2, 20–693 pg/ml; and progesterone (PGR), 0.15–28 ng/ml. Other eligibility criteria included an Eastern Cooperative Oncology Group (ECOG) performance status of 0–1 and adequate baseline bone marrow function (absolute neutrophil count, >1500/ml; platelet count, >100,000/ml), hepatic function [serum bilirubin, <2.0 mg/dl; level of transaminase: aspartate aminotransferase (AST); alanine aminotransferase (ALT), <5 times the upper normal institutional limit] and renal function (creatinine, <1.4 mg/dl). Patients with low-risk BC (papillary, medullary or mucinous), metastases or malignancies other than curatively treated skin and cervical cancer were excluded. This phase II study was performed in accordance with the Declaration of Helsinki and the European Union Guidelines on Good Clinical Practice, and was approved by the ethical committees of the participating institutions. Written informed consent was obtained from each patient.

### Treatment plan

Immediately after surgery and throughout the 5 years, patients received psychological support to improve compliance to treatment. Starting two weeks after surgery, patients were administered 11.25 mg of an LH-RH analogue by deep intramuscular injection every 12 weeks for 5 years (11). Chemotherapy was started two weeks after the LH-RH analogue and was tailored to the intrinsic characteristics of each tumour. All patients received epirubicin and docetaxel (75 mg/m^2^ each) every three weeks for four courses. Radiation therapy (XRT) was delivered at a total dose of 5000 cGy (200 cGy/fraction, five fractions/week) to the chest wall after mastectomy or to the residual breast after breast-conserving surgery and to the apex of the axilla and supraclavicular lymph nodes. A boost of 1000 cGy was delivered to the tumour bed. Concurrent with XRT, four courses of cyclophosphamide (600 mg/m^2^), methotrexate (40 mg/m^2^) and 5-fluorouracil (600 mg/m^2^; CMF) were administered, on day 1 every 3 weeks. At one month after completion of chemo-radiation therapy, the 17 patients with triple-negative disease received two courses of dose-dense chemotherapy (HDCT) with carboplatin (AUC=7), etoposide (400 mg/m^2^), ifosfamide (6000 mg/m^2^) and uromitexan (6000 mg/m^2^) over three days, supported by glycosylated recombinant granulocyte colony-stimulating factor (G-CSF; 300 μg/day), without peripheral blood progenitor cell support (12,13). Starting at one month after completion of chemotherapy or chemo-radiation therapy and continuing for five years, patients with ER^+^ tumours received hormonal therapy. After adjuvant treatment with trastuzumab was approved in 2006, patients with human epidermal growth factor receptor 2-positive (CERB-2^+^) tumours received this monoclonal antibody for one year.

### Marker evaluation

All marker assays were performed on serum and blood samples at baseline (two weeks after surgery), after chemotherapy and every year. Serum VEGF values were expressed as pg/ml and were analysed with a Colorimetric ELISA kit (Pierce Endogen, Pittsburgh, PA, USA) as previously described (14). T-Regs were assayed by flow cytometry. Plasma levels of 17β-E2, FSH, LH and PGR were evaluated by enzyme immunoassay (Tosoh Corporation, Tokyo, Japan).

### Statistical analyses

The primary endpoint of the study was the assessment of VEGF and T-Regs before and after adjuvant therapy. The number of patients required for the study was calculated according to a Simon minimax design (15). The first stage required that ≥21 of 34 patients demonstrated confirmed decreases of VEGF and T-Regs to rule out an undesirably low response probability of 0.1 (P0), and to achieve a desirable probability of 0.30 (P1), with a 5% probability of accepting a poor agent (α=0.05) and a 20% probability of rejecting a good agent (β=0.20). In the second stage, up to a total of 100 assessable patients could be added, if ≥64 patients showed confirmed VEGF and T-Regs decreases, meeting the primary endpoint. The results of laboratory tests are expressed as the mean ± standard deviation of four measurements, and the differences were determined using a repeated-measure analysis of variance. The secondary endpoints were PFS (defined as the time between the start of therapy to any relapse or the appearance of a second primary cancer or mortality, whichever occurred first) and OS measured from study entry to mortality, or 31 July 2012 for censored patients. PFS was analysed using the Kaplan-Meier method (16). All comparisons were performed using Pearson’s χ^2^ contingency table analysis. Statistical analysis was performed with SAS statistical software (version 8.12, 2000; SAS Institute Inc., Cary, NC, USA).

## Results

### Patient characteristics

A total of 100 women were recruited between April 2003 and July 2008. The median age of the patients was 43 years (range, 26–45). Table I lists their mean baseline characteristics. A total of 75 patients were treated with breast-conserving surgery, while 25 underwent modified radical mastectomy. The median number of positive axillary nodes was 3.3. A total of 83 patients were ER^+^ and 17 patients had triple-negative disease. The median Ki-67 was 33%. Of the 20 patients with CERB-2^+^ tumours, 13 were treated with trastuzumab after 2006; seven were not since their treatment occurred before its approval as adjuvant therapy. All patients were included in the analysis according to the intent-to-treat principle.

### Treatment efficacy

After a median follow-up of 70 months (minimum, 4 years), all patients were evaluable for VEGF and T-Regs. Table II shows all marker variations during the study period. The mean VEGF value at two weeks after surgery was 421±75 pg/mm^3^. After 1, 2 and 4 years, the values were 134±29, 88±12 and 55±21 pg/mm^3^, respectively. The baseline T-Reg count was 115±20 mm^3^; after 1, 2 and 4 years it was 84±14, 54±21 and 35±18 mm^3^, respectively. The mean baseline E2 and PGR serum concentrations were 95.9±6 and 1.61±03 pg/ml, respectively. After one yeat these dropped to <40 and <0.5 pg/ml, respectively, and remained low for the rest of the study period. Similarly, the mean baseline values of FSH and LH were 14.9±1.7 and 8.4±1 mU/ml, respectively. After one year they dropped to 3.14±0.3 and 0.37±0.2 mU/ml, respectively, and remained low with small variations for the remaining study period. After a mean time of 8 months from the end of the therapy, 35 (55%) of the 63 patients that had finished the 5-year treatment resumed normal menses and showed normal levels of E2, PGR, FSH and LH. Three full-term pregnancies and one voluntary abortion were reported in the women that had completed treatment. After a median follow-up period of 70 months (minimum, 4 years), PFS and OS were 94 and 98%, respectively. There was no statistically significant difference between the PFS of ER^+^ and ER- patients (P=0.08). Six patients suffered with recurrences (3 ER^+^, 3 ER^−^) after a median time of 31.3 months: Three loco-regional, two visceral (brain, lung) and one case of bone metastasis. Three patients with recurrence had >10 axillary nodes and three were CERB-2^+^, not treated with trastuzumab. No recurrence was observed after 4 years of follow-up.

### Toxicity

The grades of the adverse events are summarised in Table III. Toxicity grading was performed according to NCI-common terminology criteria for adverse events version 3.0 (CTCAE). The delivered dose intensities were as follows: 95% for docetaxel and epirubicin, 98% for CMF and 98% for high-dose chemotherapy. With LH-RH analogues, 90% of patients complained of hot flushes, mood modification and vaginal dryness. Bone mineral density was assessed at baseline and annually thereafter. The median T-score was −1.3 (range, −2, +1.5) at baseline and −2.0 (range, −4, −1.2) at the fifth year after LH-RH analogue treatment. Arthralgias and muscle weakness were common, but did not interfere with activities of daily life. With anthracycline chemotherapy, grade 3–4 haematological toxicity was observed in 41 patients, gastrointestinal toxicity (diarrhoea and mucositis) in 22 patients and severe nausea and vomiting in 19 patients. Grade 2 fever was reported in 2 patients. Grade 3 alopecia was universal. No cardiac toxicity was observed and no patient showed a significant reduction in left ventricular ejection fraction. In CMF chemotherapy, no unexpected toxicity emerged and all patients completed the scheduled treatment. Three patients experienced grade 3 haematological toxicity and diarrhoea that led to XRT interruption for one week. Thirteen patients reported grade 3 nausea and emesis. Grade 2 hepatic toxicity was observed in five patients.

In dose-dense chemotherapy, nausea and vomiting were observed in 6 instances, but were of mild intensity due to the appropriate use of ondansetron and dexamethasone. Grade 3–4 neutropenia and thrombocytopenia were observed in all patients. An absolute neutrophil count of <5x10^3^/ml was observed for a median of 4.5 days (range, 3–5 days) and a platelet count of <50x10^3^/ml occurred for a median of one day (range, 0–3 days). No patient required a platelet transfusion. Anaemia was infrequent due to the use of erythropoietin and only occurred in 13 patients (13%). Three patients had a fever of >38°C for a median duration of three days (range, 0–6) days. Grade 2 mucositis occurred in four patients and grade 3 diarrhoea in three patients. One patient had a documented infection with a positive blood culture for *Staphylococcus epidermidis*. Bone pain was reported by 2 patients, with a median duration of two days. There was no treatment-related mortality.

## Discussion

Advances in medical oncology, including several approaches for adjuvant treatment, have improved the prognosis of premenopausal patients with BC; however, young women with metastatic deposits in axillary nodes show a 10-year survival rate as low as 57.9% (17). Ovarian ablation, among the oldest described methods for treating advanced BC (18), has been confirmed by meta-analyses to be effective in the adjuvant treatment of premenopausal patients (19,20). With the development of new cytotoxic drugs, enthusiasm for the use of chemotherapy has grown. The Early Breast Cancer Trialists’ Collaborative Group showed a highly significant benefit from chemotherapy compared with no chemotherapy in the adjuvant treatment of BC, especially for women of <50 years of age (21). However, due to the poor results obtained in certain sets of patients (17), the improvement of our knowledge and the need for less toxic treatments, the value of hormonal therapy has continued to grow.

The importance of oestrogens in BC development has been highlighted by several studies. The Nurses’ Health Study II evaluated 18,521 premenopausal women and showed that the highest follicular total and free E2 levels were associated with significantly increased BC risks (22). Young age has been reported as an independent predictor of worse survival in patients with BC; this correlation may be correlated with higher levels of circulating oestrogens (23), which activate an angiogenic switch promoting further tumour progression (24).

Therefore, our primary objective was to decrease circulating oestrogens. Through administration of an LH-RH analogue, this goal was rapidly reached and maintained for five years.

Two weeks after LH-RH analogue administration, chemotherapy was started. All patients were initially treated with anthracyclines and taxanes, as this combination has been shown to improve the PFS and OS of high-risk early BC patients, independent of tumour and patient characteristics (25). Subsequently, concurrent chemotherapy and radiation therapy were delivered over eight weeks, which is reportedly associated with a lower risk of local recurrence (39% decrease) in node-positive patients (26). The sequential administration of LH-RH analogue and chemotherapy was designed to compensate for tumour heterogeneity; oestrogen deprivation may induce apoptosis on slowly proliferating cell clones that are not sensitive to chemotherapy (27), while chemotherapy may be active in cellular clones that are rapidly proliferating.

VEGF is an important prognostic factor for PFS and OS in primary node-positive and node-negative BC, and it may be useful in predicting hormone unresponsiveness of ER^+^ patients (28–30). In addition, it has been shown that 70% of patients with advanced BC have high serum VEGF (31). High plasma VEGF has been associated with progressive advanced-stage disease, and in several studies VEGF level changes were correlated with treatment response (8,9).

Another action of VEGF is the increase in vascular permeability that may augment tumour cell extravasation and metastasis formation. Moreover, VEGF may be responsible for the decreased immune competence observed in advanced cancer, through the reduced maturation of dendritic cells that are important antigen-presenting cells (32). For this reason, prolonged exposure of the immune system to high levels of VEGF may decrease immune response and, thus, facilitate tumour growth. Moreover, VEGF upregulates BCL-2 and inhibits apoptosis in human and murine mammary adenocarcinoma cells (33).

T-Regs are also associated with prognosis and progression in invasive and noninvasive BC, and are an independent molecular marker for BC clinical outcome (10,34). Furthermore, the numbers of T-Regs were higher in patients with invasive ductal carcinomas compared with invasive lobular cancers (35). Additionally, a positive correlation has been shown between FOXP3 and VEGF expression (36).

Both of our primary endpoints were met: Statistically significant decreases in levels of VEGF and T-Regs were observed at four years.

The five-year PFS and OS rates were 93.4 and 98%, respectively. Although our study was a non-randomised phase II study, the patient accrual was consecutive for five years, with no selection bias. Only high-risk patients were accrued; 31 patients with lower risk were screened but not entered into the study (data not shown). In contrast to other studies, our protocol planned the administration of LH-RH analogues from the beginning of therapy and concurrent with chemotherapy, XRT. The analogue was administered to all patients, and chemotherapy was tailored to each patient’s characteristics. The ZEBRA adjuvant trial enrolled premenopausal patients with node-positive BC, similar to our population in age distribution and ER status, and randomised patients to CMF or LH-RH analogue (37). ZEBRA patients randomised to CMF who were amenorrhoic at 36 weeks showed a significant improvement in PFS compared with those who were not, demonstrating that the endocrine effect of chemotherapy may be due to oestrogen deprivation, which was achieved in all patients in our study. A recent analysis of the National Surgical Adjuvant Breast and Bowel Project Protocol B-30 trial has revealed that amenorrhoea after chemotherapy was correlated with a substantial survival advantage (38). In addition, another just published study has shown that persistence of menstruation after cytotoxic chemotherapy was a poor prognostic factor for disease-free survival in premenopausal patients with early BC (39).

In conclusion we have shown that E2 deprivation with an LH-RH analogue, followed by tailored chemotherapy, radiation therapy and hormonal therapy in ER^+^ tumours, decreased plasma VEGF and T-Regs in premenopausal high-risk ER^+^ and ER^−^ BC patients.

## Figures and Tables

**Figure 1:**
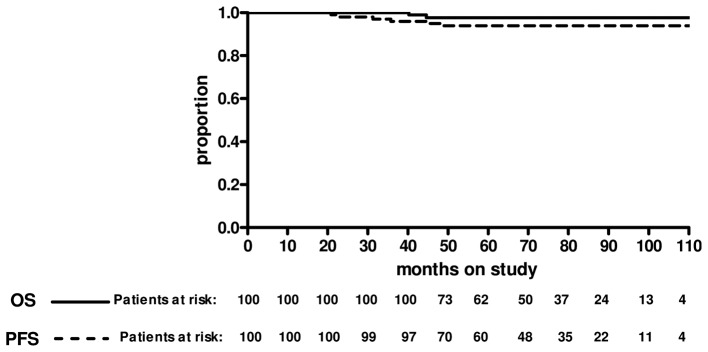
Progression-free survival (PFS). Events, 7 (7%); censored, 93 (93%). The five-year PFS rate was 94%. Overall survival (OS): Events, 2 (3%); censored, 98 (98%). The five-year OS rate was 98%. The median follow-up was 70 months.

**Table I:** Patient and tumour characteristics.

Characteristics	Value
No. of patients	100
Age, years	
Median	43
Range	26–45
Hormone receptors status, n=%	
ER^+^	83
ER^–^	17
Tumour histology, n=%	
Ductal infiltrating	88
Lobular infiltrating	6
Undifferentiated	6
Grading, n=%	
G1–G2	66
G3	34
Clinical stage, n=%	
IIA	62
IIB	13
IIIA	11
IIIB	4
IIIC	10
Positive axillary nodes, n=%	
1–3	79
4–9	11
>10	10
Type of primary surgery, n=%	
Mastectomy	25
Quadrantectomy	75

ER, oestrogen receptor.

**Table II:** Marker variation.

Time	VEGF (pg/ml)	T-Regs (n/ml)	Estradiol (ng/ml)	PGR (ng/ml)	FSH (mU/ml)	LH (mU/ml)
Baseline (100 patients)	421±75	115±20	95.9±6	1.61±03	14.9±1.7	8.4±1
1 year	134±29	84±14	8.34±1.2	0.4±0.2	3.14±0.3	0.37±0.2
(100 patients)	P<0.0001	P<0.0001	P<0.0001	P<0.0001	P<0.0001	P<0.0001
2 years	88±12	54±21	5.3±0.8	0.32±1.1	2.65±0.4	0.32±0.2
(100 patients)	P<0.0001	P<0.0001	P<0.0001	P<0.0001	P<0.0001	P<0.0001
4 years	55±21	35±18	5.7±0.7	0.44±1.1	2.14±0.4	0.25±0.2
(100 patients)	P<0.0001	P<0.0001	P<0.0001	P<0.0001	P<0.0001	P<0.0001

Values are presented as mean ± SD. VEGF, vascular endothelial growth factor; T-Regs, T-regulatory cells; FSH, follicle-stimulating hormone; LH, luteneizing hormone; PGR, progesterone. P-values vs. baseline value.

**Table III:** Toxicity (grade 3–4).

Adverse events	Type of therapy			
	LH-RH analogue (n=100), n=%	Anthracycline-taxanes (n=100), n=%	CMF+XRT (n=100), n=%	HDCT (n=17), n (%)
Haematological				
Leukopenia	0	32	3	17 (100)
Thrombocytopenia	0	9	0	17 (100)
Anaemia	0	9	0	4 (23)
Gastrointestinal				
Nausea-vomiting	0	19	13	6 (35)
Diarrhoea	0	11	3	3 (17)
Mucositis	0	11	8	0
Infection	0	2	0	3 (17)
Neurotoxicity grade 2	0	15	0	0
Alopecia	0	100	0	17 (100)
Hot flushes	90	0	0	0

CMF, cyclophosphamide, methotrexate, 5-fluorouracil; XRT, radiation therapy; HDCT, dose-dense chemotherapy; LH-RH, luteneizing hormone-releasing hormone.
